# Generalizing game-changing species across microbial communities

**DOI:** 10.1038/s43705-021-00022-2

**Published:** 2021-06-08

**Authors:** Jie Deng, Marco Tulio Angulo, Serguei Saavedra

**Affiliations:** 1 Department of Civil and Environmental Engineering, MIT, Cambridge, MA USA; 2 CONACyT – Institute of Mathematics, Universidad Nacional Autónoma de México, Juriquilla, México

**Keywords:** Microbial ecology, Microbial ecology, Theoretical ecology, Community ecology

## Abstract

Microbes form multispecies communities that play essential roles in our environment and health. Not surprisingly, there is an increasing need for understanding if certain invader species will modify a given microbial community, producing either a desired or undesired change in the observed collection of resident species. However, the complex interactions that species can establish between each other and the diverse external factors underlying their dynamics have made constructing such understanding context-specific. Here we integrate tractable theoretical systems with tractable experimental systems to find general conditions under which non-resident species can change the collection of resident communities—*game-changing* species. We show that non-resident colonizers are more likely to be game-changers than transients, whereas game-changers are more likely to suppress than to promote resident species. Importantly, we find general heuristic rules for game-changers under controlled environments by integrating mutual invasibility theory with in vitro experimental systems, and general heuristic rules under changing environments by integrating structuralist theory with in vivo experimental systems. Despite the strong context-dependency of microbial communities, our work shows that under an appropriate integration of tractable theoretical and experimental systems, it is possible to unveil regularities that can then be potentially extended to understand the behavior of complex natural communities.

## Introduction

Microbes form multispecies communities (microbiota) that play essential roles in maintaining our environment and health, from regulating natural resources and balancing climatic factors, to stimulating our immune system and protecting us from pathogens.^1–4^ These microbial communities can be modified by *non-resident* microbes (i.e., species not originally in the pool of residents), producing desired or undesired changes in the observed collection of resident species,^4,5^ For example, in the human gut microbiota, non-resident species can modify the resident community leading to changes associated with chronic gastrointestinal diseases,^6^ or they can produce changes that cure their hosts of recurrent infections,^7,8^ Indeed, via interventions like probiotic cocktails or microbiota transplants, there is a growing interest in regulating microbial communities by promoting or avoiding the introduction of *colonizing* (species that can become established in a perturbed community) and *transient* (species that cannot colonize) non-resident species.^9–12^

However, explaining and predicting if a non-resident species will modify or not a given microbial community has become a context-specific problem.^13–17^ These challenges originate from the intricate interactions that species can establish between each other,^18–20^ together with the diverse external conditions underlying the dynamics of microbial communities.^21–24^ Importantly, *synthetic (microbial) ecology* has emerged as a promising framework to formalize model systems and address the challenges above by either systematically modifying the collection of species, altering abiotic conditions, or using genome-based technologies.^25–27^ In particular, it has been suggested that the appropriate integration of tractable mathematical and experimental systems can provide a general system-level causative knowledge about the dynamics of entire microbial communities (not just single species—the realm of synthetic biology).^26,27^ That is, tractable theoretical models can allow us to understand the possible solutions of a system and build predictions that can then be corroborated and replicated using tractable experimental systems. In this line, both in vitro and in vivo communities have emerged as tractable experimental systems^4,26^ that can be used to study community dynamics under controlled and changing environmental conditions, respectively. Yet, while tractable theoretical systems have a long tradition in microbial ecology,^26–28^ it remains unclear which of these theoretical and experimental systems can be integrated to answer how and when it is expected that a non-resident species modify a given resident community.

To address the generalization problem defined above, we investigate whether tractable theoretical systems can be integrated with in vitro and in vivo microbial communities to find potential regularities characterizing *game-changing* species across communities—transient or colonizing non-resident species that can promote or suppress the establishment of resident species. First, using in vitro communities formed by soil bacteria and in vivo communities formed by gut bacteria of *Drosophila melanogaster* fruit fly, we study the extent to which colonizing and transient non-resident species have different probabilities of being game-changers under controlled and changing environmental conditions. In particular, we investigate whether there is an intrinsic capacity of individual species to change resident communities or it is all community-specific. Second, we investigate the extent to which heuristic rules based on mutual invasibility theory,^29,30^ and structuralist theory,^31,32^ can be integrated with the experimental systems to find generalities for game-changers across communities. Lastly, we discuss the implications of our results towards their extensions to complex natural communities.

## Methods

### Tractable systems

**Fig. 1:**
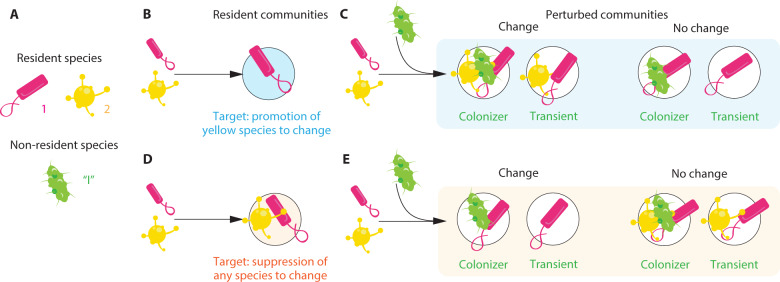
Game-changing species for a resident microbial community. Illustration of different contexts leading to game-changing and non-game-changing species. Panel **A** shows a hypothetical microbial community with a pool ${\mathcal{R}} =\{1,2\}$ of two resident species (pink and yellow) and one non-resident species “$I$” (green). Panel **B** shows the context when one species excludes the other, the resident community contains a single resident (${\mathcal{M}} =\{1\}$). To change the resident community, the non-resident species needs to promote the establishment of the other species (in this case yellow, but the example can also be done for the pink species). Panel **C** provides examples of game-changing and non-game-changing non-resident species for the example presented in Panel **B** (as the outcomes of the perturbed communities ${ {\mathcal{M}} }_{p}$). Panel **D** shows another context when the two resident species coexist (${\mathcal{M}} =\{1,2\}$). To change the resident community, the non-resident species needs to suppress the establishment of any of the species (green or yellow). Panel **E** provides potential outcomes of perturbed communities of game-changing and non-game-changing non-resident species for the example presented in Panel **D**. In this context, the change happens by suppressing the yellow species (the same can be said for the pink species). Note that in all contexts, the non-resident species can be either a colonizer (can become established in the perturbed community) or transient (cannot colonize).

Tractable systems are models typically of known and reduced complexity that can be operationalized and reproduced over relatively short periods of time. To formalize our study using tractable systems, we consider a (regional) pool ${\mathcal{R}} =\left\{1,2,\cdots ,S\right\}$ of $S$ *resident* species and one *non-resident* species denoted by “$I$” (Fig. 1A). We denote the *resident community* by ${\mathcal{M}} \subseteq {\mathcal{R}}$, which is the species collection that coexists obtained by assembling all resident species simultaneously. Additionally, let ${ {\mathcal{M}} }_{p}$ denote the *perturbed community* formed by the species collection that coexists when assembling all residents and the non-resident species simultaneously assuming the possibility of multiple introductions. Note that this mechanism corresponds to a top-down assembly process.^33^ Then, the non-resident species is classified as a game-changing species if it changes the number of resident species that coexist: $|{ {\mathcal{M}} }_{p}\{I\}|\,\ne\, | {\mathcal{M}} |$. Figure 1A illustrates the concept of a game-changing species in a hypothetical microbial community of $S=2$ resident species. Here, one possible context for the resident species is that one species excludes the other, e.g., ${\mathcal{M}} =\{1\}$ (Fig. 1B). In this case, the non-resident species is a game-changing species if it promotes the establishment of the other resident (Fig. 1C). Note that we do not consider a change when eliminating the current resident species. The other context for the resident species is that both coexist ${\mathcal{M}} =\{1,2\}$ (Fig. 1D). In this case, a non-resident species is a game-changing species if it suppresses the establishment of at least one of the residents, e.g., ${ {\mathcal{M}} }_{p}=\{1,I\}$ (Fig. 1E). Note that a game-changing species can be either colonizer or transient depending on the dynamics.

We study the generalization of game-changing species under controlled conditions using in vitro experimental soil communities and under changing conditions using in vivo gut microbial communities (see SI for details about these experimental systems). Note that in vitro experiments usually create ad hoc conditions for species by putting them outside of their natural changing habitats, assuring that species survive in monocultures, and by forming interspecific interactions that may not occur otherwise. Instead, in vivo experiments are performed within living systems, resembling much closer the natural habitat of species and their interspecific interactions. Both types of systems are of reduced complexity, allowing the monitoring and reproducibility of experiments. The studied in vitro soil communities are formed by experimental trials of eight interacting heterotrophic soil-dwelling microbes:^30^ *Enterobacter aerogenes*, *Pseudomonas aurantiaca*, *Pseudomonas chlororaphis*, *Pseudomonas citronellolis*, *Pseudomonas fluorescens*, *Pseudomonas putida*, *Pseudomonas veronii*, and *Serratia marcescens*. These experiments were performed by co-inoculating species at different growth–dilution cycles into fresh media. Each species was cultured in isolation. All experiments were carried out in duplicate. The studied in vivo gut communities are formed by experimental trails of five interacting microbes commonly found in the fruit fly *Drosophila melanogoster* gut microbiota:^34^ *Lactobacillus plantarum*, *Lactobacillus brevis*, *Acetobacter pasteurianus*, *Acetobacter tropicalis*, and *Acetobacter orientalis*. These experiments were performed by co-inoculating species through frequent ingestion in different flies. All experiments were replicated at least 45 times. These two data sets are, to our knowledge, the closest and best described systems of two- and three-species communities currently available describing species coexistence (not just presence/absence records) under two contrasting environmental conditions.

Focusing on in vitro communities, we studied all 28 pairs and 56 trios formed by the eight soil species.^30^ This provided 168 cases, where it is possible to investigate the expected result (${ {\mathcal{M}} }_{p}$) of assembling a non-resident species together with a resident community (21 cases for each of the 8 studied microbes). The overall competition time was chosen such that species extinctions would have sufficient time to occur, while new mutants would typically not have time to arise and spread. Similarly for the in vivo communities, we studied all 10 pairs and 10 trios formed by the 5 gut species, which provided 30 cases equivalent to the soil experiments.^34^ Because species extinctions in in vivo communities are harder to establish, we classified as an expected extinction to any species whose relative abundance was less than 10% in at least 71% of all (47–49) replicates, which corresponds to less than 1% of cases under a binomial distribution with $p=0.5$ (slightly different thresholds produce qualitatively similar results). Each of these 168 and 30 cases for soil and gut communities, respectively, represents a given resident community (${\mathcal{M}}$) formed by a pool of two resident species (${\mathcal{R}} =\{1,2\}$, ${\mathcal{M}} \subseteq {\mathcal{R}}$) where the target for a non-resident species ($I$) can be either to promote or to suppress the establishment of resident species (${\mathcal{R}} {\cup }\left\{I\right\}$, ${\mathcal{M}}_{p} \setminus \{I\}\,\ne\, {\mathcal{M}}$). Non-resident species that are expected to survive in ${ {\mathcal{M}} }_{p}$ are classified as colonizers; otherwise they are classified as transients.

### Empirical contexts

To investigate the role of context dependency in the game-changing capacity of microbial species, we study the extent to which a given species can be classified as a game-changer regardless of the resident community it interacts with or if it is the resident community that provides the opportunity for a non-resident species to be a game-changer. Specifically, for each species, we calculate the fraction of times such a species changes the resident community conditioned on the type (whether it is a colonizer or a transient) and target (whether promoting or suppressing). Then, we calculate the probability ($p$ value) of observing a fraction greater than or equal to the observed fraction under the given type/target (using a one-sided binomial test with mean value given by the empirical frequency within each type/target). High $p$ values (e.g., $> 0.05$) would be indicative of the importance of context-dependency and not of the intrinsic capacity of species.

Next, we quantify the average effect of empirical contexts shaping the game-changing capacity of non-resident species. Specifically, we measure the *type’s average effect* on changing the community using ${E}_{Y}=P(C=1|Y=1)-P(C=1|Y=0)$. Here, $C=1$ if the non-resident species was a game-changer ($C=0$ if it was not), and where $Y=1$ if the species was a colonizer ($Y=0$ if the species was transient). The non-parametric quantity $P(C|Y)$ corresponds to the frequency of observing $C$ given $Y$. Thus, ${E}_{Y} \,> \, 0$ (resp. ${E}_{Y} \,<\, 0$) indicates that a game-changing species is more likely to be a colonizer (resp. transient). Similarly, we measure the *target’s average effect* on changing the community using ${E}_{T}=P(C=1|T=1)-P(C=1|T=0)$, where $T=1$ if the target was to suppress ($T=0$ if the target was to promote) and $P(C|T)$ corresponds to the frequency of observing $C$ given $T$. Thus, ${E}_{T} \,> \, 0$ (resp. $\,{E}_{T} \,<\, 0$) indicates that a game-changing species is more likely to suppress (resp. promote) the establishing of resident species. High effects (and statistically different from what would be expected by chance using a G${}^{2}$-test) would be indicative of the impact of the empirical contexts on the game-changing capacity of species.

### Tractable theoretical systems: mutual invasibility theory

**Fig. 2:**
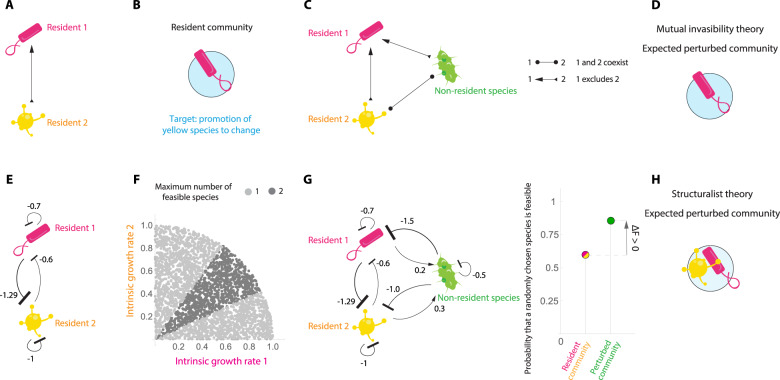
Heuristc rules for the generalization of game-changing species. As an example, Panel **A** illustrates a pool of resident species, where Resident 1 tends to exclude Resident 2. Panel **B** shows the resulting resident community ${\mathcal{M}} =\{1\}$, where the target for a non-resident species is to promote Resident 2. Mutual invasibility theory: Panel **C** illustrates an example of experimental information needed to generate the heuristic rule based on mutual invasibility. According to this rule the non-resident species (green) will be a game-changer if Resident 2 can survive in every single pair. Panel **D** shows that the non-resident species will not be able to change the community based on mutual invasibility. Structuralist theory: Panel **E** shows an example of experimental information needed to generate the heuristic rule based on structuralist theory. Arrows indicate the values of the interspecific and intraspecific interactions. According to this rule the non-resident species (green) will be a game-changer if it can increase the probability of feasibility. In Panel **F**, each dot is a value of the external factors ${\boldsymbol{\theta }}=({\theta }_{1},{\theta }_{2})$, representing the effect of external conditions (e.g., intrinsic growth rates in a Lotka-Volterra system). Values are chosen uniformly at random over the positive quadrant of the unit ball. Depending on the value of these two intrinsic growth rates, the theoretical maximum number of feasible species varies between one (light gray) and two (dark gray) species following a linear Lotka-Volterra system. Panel **G** shows the non-resident species (green) and its corresponding pairwise effects on the resident species. By adding the non-resident species (right green dot), the probability that a randomly chosen species is feasible can either increase or decrease compared to the probability without the non-resident species (left pink/yellow dot). In this example, the non-resident species increases the probability of feasibility (${\Delta }_{{\rm{F}}} \,> \,0$). Panel **H** shows that the non-resident species will be able to change the community based on structuralist theory.

We use tractable theoretical systems to establish sufficiently operationalizable algorithms that can move us away from context-specific cases to regularities shaping the capacity of game-changing species. The first premise follows a heuristic assembly rule based on mutual invasibility theory.^30^ Mutual invasibility theory has been a widely-adopted tractable premise in ecology and evolution,^29,35^ This theory states that in a multispecies community, species that all coexist with each other in sub-communities will survive, whereas species that are excluded by any of the surviving species will go extinct^30^ (Fig. 2A–D). Despite its strict assembly requirements, this heuristic rule has proved relatively successful in predicting the outcome of surviving species in the studied in vitro soil communities.^30^ Thus, to operationalize this assembly rule, we introduce a binary variable (${{V}}$) that when anticipating the promotion (resp. suppression) of resident species becomes ${{V}}=1$ (resp. ${{V}}=0$) if and only if all the resident species survive when paired with any other species; otherwise ${{V}}=0$ (resp. ${{V}}=1$). Then, we measure the expected effect of mutual invasibility on the capacity of game-changing species while keeping all other factors constant at whatever value they would have obtained under a non-invasibility case—known as the direct natural effect:^36^ ${{\rm{NE}}}_{{{V}}}={\Sigma }_{{{T}},{{Y}}}[P(C=1|{{V}}=1,\,T,Y)-P(C=1|{{V}}=0,T,Y)]P(T,Y|{{V}}=0)$. Positive effects (and statistically different from what would be expected by chance using a G${}^{2}$-test) would be indicative of the usefulness of this heuristic rule as a general context for identifying game-changing species.

### Tractable theoretical systems: structuralist theory

While mutual invasibility theory has provided key insights regarding population dynamics,^29^^,^^30^^,^^35^ it has been shown that it cannot be directly generalized to multispecies communities.^37–39^ Hence, as an alternative potential generalization, we introduce a second heuristic rule based on structuralist theory,^32,40,41^ Across many areas of biology, the structuralist view has provided a systematic and probabilistic platform for understanding the diversity that we observe in nature,^31,42,43^ In ecology, structuralist theory assumes that the probability of observing a community is based on the match between the internal constraints established by species interactions (treated as physico-chemical rules of design) within a community and the changing external conditions (treated as unknown conditions).^32,44,45^ This other premise has also been shown to be as successful as mutual invasibility in predicting the outcome of surviving species in the studied in vitro soil communities,^32^ but it has not been tested for its generality.

Formally, the structuralist framework assumes that the per-capita growth rate of an $i$th species can be approximated by a general phenomenological function ${f}_{i}({N}_{1},\cdots ,{N}_{S},{N}_{I};{\boldsymbol{\theta}})$, i.e.,

1 $$\frac{d{N}_{i}}{dt}={N}_{i} {f}_{i}({N}_{1},\cdots ,{N}_{S},{N}_{I};{\mathbf{\theta}}),\qquad i\in {\mathcal{R}}{\cup}\{I\}$$

Above, ${N}_{i}$ represents the abundance (or biomass) of species $i$. The functions ${f}_{i}$ encode the internal constraints of the community dynamics.^46^ The vector parameter ${\mathbf{\theta}}$ encodes the external (unknown) conditions acting on the community, which can change according to some probability distribution $p({\mathbf{\theta}})$. For a particular value ${\mathbf{\theta}}={{\mathbf{\theta}}}^{\ast }$, a species collection ${\mathscr{Z}}\subseteq {\mathcal{R}} {\cup }\{I\}$ is said *feasible* (potentially observable) for Eq. (1) if there exists equilibrium abundances ${N}_{i}^{\ast } \,> \, 0$ for all species $i\in {\mathscr{Z}}$ and ${N}_{i}^{\ast }=0$ for $i\,\notin\, {\mathscr{Z}}$ (i.e., ${f}_{i}({N}_{1}^{\ast },\cdots ,{N}_{S}^{\ast },{N}_{I}^{\ast };{{\mathbf{\theta}}}^{\ast })=0$ for all $i$).^40^ Then, we can use Eq. (1) to push-forward $p({\mathbf{\theta}})$ and estimate the probability that a randomly chosen species $i$ is feasible with ($i\in {\mathcal{R}} {\cup }\{I\}$) and without ($i\in {\mathcal{R}}$) the non-resident species under isotropic changing conditions, respectively. In this form, the effect of a non-resident species $I$ on a resident community can be characterized by the expected *maximum* impact on its feasibility, i.e., ${\Delta }_{{{F}}}=p(i| {\mathcal{R}} {\cup }\{I\})-p(i| {\mathcal{R}} )$ (Fig. 2E–H).

To make this framework tractable, we leverage on the mathematical properties of the linear Lotka–Volterra (LV) system^47^ with the per-capita growth rate ${f}_{i}({N}_{1},\cdots ,{N}_{S},{N}_{I};{\mathbf{\theta}})=\mathop{\sum}\limits_{j\in {\mathcal{R}} {\cup }\{I\}}{a}_{ij}{N}_{j}+{\theta}_{i}$ for $i\in {\mathcal{R}} {\cup }\{I\}$. While the linear LV system can be interpreted under many different assumptions,^47^ we follow its most general interpretation as a first-order approximation to Eq. (1).^46^ In this system, the time-invariant community structure consists of the intraspecific and interspecific species interactions ${\bf{A}}=({a}_{ij})\in {{\mathbb{R}}}^{(S+1)\times (S+1)}$, and the external factors ${\mathbf{\theta}}=({\theta }_{1},\cdots ,{\theta }_{S},{\theta }_{I})\in {{\mathbb{R}}}^{S+1}$ consist of density-independent intrinsic per-capita growth rates of all species. We assume that $p({\boldsymbol{\theta }})$ is uniform over the positive parameter space (conforming with ergodicity in dynamical systems^32^) and find analytically the external conditions compatible with the feasibility of a randomly chosen species within a given community ${\bf{A}}$, i.e., $p(i|{\bf{A}})$ (see SI). This framework is robust to changes in the system dynamics since $p(i|{\bf{A}})$ is identical for all systems that are topologically equivalent to the linear LV system^32^^,^^48^ and a lower bound for systems with higher-order terms.^41^ Note also that while higher-order interactions may impact the dynamics of microbial communities^49^^,^^50^, their incorporation into ecological models as higher-order polynomials rend intractable and super-sensitive systems (no closed-form solutions can be found in terms of radicals)^41^^,^^51–53^.

To quantify the contribution to feasibility (${\Delta }_{{\rm{F}}}$) of a non-resident species under the structuralist framework defined above, we infer both the resident interaction matrix ${\bf{A}}$ and the perturbed interaction matrix ${{\bf{A}}}_{p}$ using only information from experimental monocultures and pairwise cocultures. Interaction matrices were inferred by fitting the linear LV system using the different repetitions of the observed survival data (see SI and Fig. S1). To make the structuralist framework comparable with the mutual invasibility framework, we introduce a heuristic rule based on structuralist theory formalized in a binary variable (${{F}}$) that when anticipating the promotion (resp. suppression) of resident species becomes ${{F}}=1$ if ${\Delta }_{{{F}}} \,> \, 0$; otherwise ${{F}}=0$ if ${\Delta }_{{{F}}} \,<\, 0$. Then, we measure the expected effect of structuralist theory on the capacity of game-changing species while keeping all other factors constant at whatever value they would have obtained under an opposite scenario:^36^ ${{\rm{NE}}}_{{{F}}}=\mathop{\sum}\limits_{{{T}},{{Y}}}[{{P}}({{C}}=1|{{F}}=1,{{T}},{{Y}})-{{P}}({{C}}=1|{{F}}=0,{{T}},{{Y}})]{{P}}({{T}},{{Y}}|{{F}}=0)$. Positive effects (and statistically different from what would be expected by chance using a G${}^{2}$-test) would be indicative of the usefulness of this heuristic rule as a general context for identifying game-changing species.

## Results

**Fig. 3:**
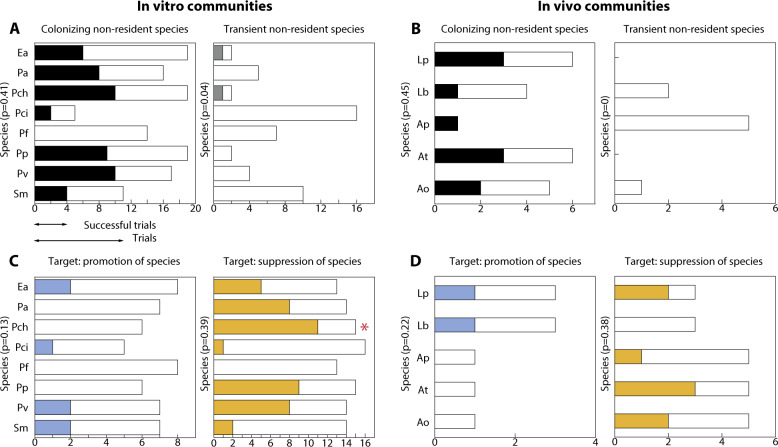
No species is a consistent game-changer. Panels **A** and **B** show for in vitro soil and in vivo gut communities, respectively, that the observed number of changes generated by each species is generally not statistically different (* = $p$ value$\,<\, 0.05$) from what can be expected by chance alone when conditioning by the type of species (colonizer or transient). Bars correspond to the number of cases a species is a colonizer (black left bars) or transient (gray right bars) when assembled together with a resident community. The colored fraction of each bar corresponds to the number of times the species changed the resident community (successful trials). Panels **C** and **D** are similar to the previous panels but conditioning by target (promote or suppress the establishment of resident species). Bars correspond to the number of cases a species is assembled together with a resident community that needs the promotion (left blue bars) or suppression (right orange bars) of resident species to change. The colored fraction of each bar corresponds to the number of changes (successful trials). Recall that the studied soil and gut communities are formed by eight and five species, respectively. Vertical axes provide the abbreviated names of species (see main text). The associated $p$ values are calculated using a one-sided binomial test $B(n,x,p)$, where $n$, $x$, and $p$ correspond to the number of total cases, number of changes, and the changing probability within the corresponding target as shown within each panel (*y*-label). The alternative hypothesis is that the probability of changing a resident community is greater than or equal to the observed number of changing cases.

In line with existing expectations,^13,17^ we corroborated that the game-changing capacity of non-resident species is not intrinsic but strongly context-dependent regardless of whether communities are subject to ad hoc (in vitro) or more natural (in vivo) environmental conditions. Specifically, the number of times an individual species changes a resident community is not significantly statistically greater than what can be expected by chance alone. In the in vitro soil communities, for only 1 out of 32 cases the $p$-value associated with the game-changing frequency was lower than 5%, whereas no single case was found in in vivo gut communities. That is, regardless of whether we conditioned by species type (Fig. 3A, B) or by target (Fig. 3C, D), the game-changing capacity of a non-resident species depends on the particular community of residents under both controlled and changing environmental conditions.

Importantly, the type and target associated with each non-resident species does provide empirical contextual information. We found that non-resident colonizers are more likely to change resident communities than transients, whereas game-changers are more likely found suppressing than promoting resident species. In particular, the average effect of species type on game-changing capacity increases ${E}_{Y}=36 \%$ with $p\;{\rm{value}} \,<\, {10}^{-3}$ (resp. ${E}_{Y}=45 \%$ with $p\; {\rm{value}} \,<\, {10}^{-3}$) when changing from a transient to colonizer non-resident in in vitro soil (resp. in vivo gut) communities. By contrast, the average effect of the target on game-changing capacity increases by ${E}_{T}=25 \%$ with $p\;{\rm{value}} \,<\, {10}^{-3}$ (resp. ${E}_{T}=16 \%$ with $p\;{\rm{value}}=0.221$) when moving from promoting to suppressing resident species in in vitro soil (resp. in vivo gut) communities.

**Fig. 4:**
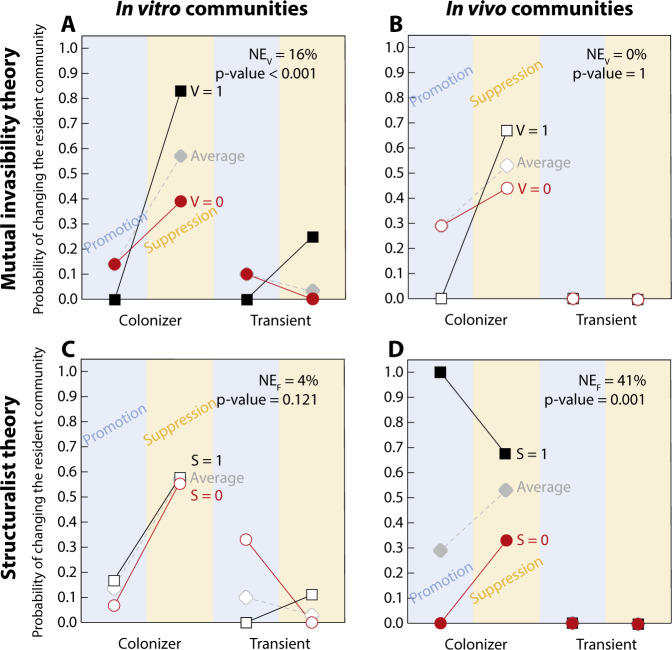
Generalizing game-changing species. Panels **A** and **B** show information based on mutual invasibility theory (${{V}}=1$, black squares; or ${{V}}=0$, red circles) for in vitro soil and in vivo gut microbial communities, respectively. The probability of non-resident species being game-changers increase by ${{\rm{NE}}}_{{\rm{V}}}=\,16 \%$ with $p\;{\rm{value}} \,<\, {10}^{-3}$ (resp. ${{\rm{NE}}}_{{{V}}}=\,0 \%$ with $p\;{\rm{value}}=1$) in in vitro soil (resp. in vivo gut) communities. Solid and open symbols correspond to statistically significant and non-significant effects, respectively. Panels **C** and **D** show information based on structuralist theory (${{F}}=1$, black squares; or ${{F}}=0$, red circles). The probability of non-resident species being game-changers increase by ${{\rm{NE}}}_{{{F}}}=4 \%$ with $p\;{\rm{value}}=0.121$ (resp. ${{\rm{NE}}}_{{{F}}}=41 \%$ with $p\;{\rm{value}} \,<\, {10}^{-3}$) in in vitro soil (resp. in vivo gut) communities. As a reference, gray diamonds connected by dashed lines correspond to the empirical context (average empirical probabilities) for the combination of being colonizer/transient under suppression/promotion of resident species.

Focusing on the potential generalization of game-changers through the integration of tractable theoretical and experimental systems, we found that the heuristic rules based on mutual invasibility theory work as a general contextualization for in vitro soil communities but not for in vivo gut communities. Instead, heuristic rules based on structuralist theory work as a general contextualization for in vivo gut communities but not for in vitro soil communities. Specifically, we found that non-resident species present in cases that fulfill the mutual invasibility theory (${\rm{V}}=1$) increase their probability of being game-changers by ${{\rm{NE}}}_{{{V}}}=\,16 \%$ with $p\;{\rm{value}} \,<\, {10}^{-3}$ (resp. ${{\rm{NE}}}_{{{V}}}=\,0 \%$ with $p\;{\rm{value}}=1$) in in vitro soil (resp. in vivo gut) communities (Fig. 4A, B). By contrast, we found that non-resident species present in cases that fulfill the structuralist theory (${{F}}=1$) increase their probability of being game-changers by ${{\rm{NE}}}_{{{F}}}=4 \%$ with $p\;{\rm{value}}=0.121$ (resp. ${{\rm{NE}}}_{{{F}}}=41 \%$ with $p\;{\rm{value}} \,<\, {10}^{-3}$) in in vitro soil (resp. in vivo gut) communities (Fig. 4C, D). Note that these heuristic rules provided significantly additional information from the empirical contexts that can be calculated to identify game-changing species without taking into account ecological theory (gray symbols in Fig. 4).

## Discussion

Identifying non-resident species that can regulate the collection of resident species in microbial communities has tremendous potential for bio-technological and bio-medical applications.^2^^,^^54^^,^^55^ However, the complex interplay among several ecological, evolutionary, and external processes^22–24^^,^^56^^,^^57^ has made unclear if it is possible to predict species achieving such regulation.^1^^,^^4^ For instance, on the one hand, there has been increasing concern about the spread of antibiotic (external perturbation) resistance among pathogens, as well as growing concern that antibiotic use may change the collection of resident species that contribute to human health.^4^ On the other hand, it has been shown that a reduction in perturbation frequency can also promote invasion by pathogens and change microbial communities.^19^^,^^58^ Thus, it has been understood that the regulation of microbial communities is a context-specific problem.^13–17^ That being said, synthetic ecology, via theoretical and experimental systems, is providing tractable platforms to unveil regularities governing the dynamics of entire microbial communities.^25–27^ Yet, a question that remains to be answered is which of these theoretical systems can be integrated with experimental systems in order to establish general causative knowledge that can be potentially extended to complex natural communities.

In this work, we have revealed that despite the strong context-specificity of microbial dynamics, it is possible to unveil regularities shaping changes in the species collection of microbial communities. Specifically, we have investigated the potential integration of two tractable theoretical systems (mutual invasibility theory and structuralist theory) with two tractable experimental systems (in vitro soil communities and in vivo gut communities) in order to find generalities for the game-changing capacity of species (non-resident species that can change the collection of resident species). Despite the fact that we have used experimental systems subject to different environmental conditions, we have found that in both in vitro soil communities and in vivo gut communities, no species is a game-changer across all communities, non-resident colonizers are more likely to change resident communities than transients, and game-changers are more likely to suppress resident species than to promote them. Importantly, although these systems have displayed similar contextual patterns for game-changing species, they have required different theoretical systems for their potential generalization.

We have found that mutual invasibility theory^30^ and structuralist theor^32^ can be used, respectively, as heuristic rules to establish additional regularities in in vitro and in vivo communities, but not in both. Although we cannot fully rule out the existence of dynamical differences between in vitro and in vivo simply due to differences in species (given the nature of the available data), it is important to note that the differences originate at the theoretical (and therefore generalization) level. That is, we have made theoretical predictions completely grounded on differences between controlled and changing conditions, and we have corroborated these predictions with experimental data. Moreover, these effects have been quantified following causal inference analysis,^36^ conditioning the effects on the potential idiosyncrasies that each experimental system harbors. This makes our results robust to small heterogeneities, but we hope future work can expand on this interesting issue following the methodological guidelines that we have introduced.

Indeed, mutual invasibility theory has been a tractable widely adopted premise in ecology, which has been successful in predicting the composition of in vitro communities.^30^ Yet, species persistence under this premise requires that species persist in all sub-communities, which can be a very stringent and ad hoc condition that may only be fulfilled in the lab.^37–39^ Instead, structuralist theory is an alternative tractable premise that assumes that dynamics are governed by a set of internal constraints (or rules of design) that have to be separated from changing external conditions.^32,59–63^ We have shown that mutual invasibility theory was able to capture regularities in in vitro communities (Fig. 4A), but not in in vivo communities (Fig. 4B). By contrast, we have found that structuralist theory provides general information for in vivo communities (Fig. 4D), but not for in vitro communities (Fig. 4C). These findings illustrate that the strict persistence requirements imposed by mutual invasibility theory may only be observed in highly controlled or ad hoc environments (such as in vitro communities), whereas the separation of internal and external factors assumed by structuralist theory may be observed in more natural conditions (such as in vivo communities).

The goal of synthetic ecology has been centered on working with tractable systems of known and reduced complexity to be able to obtain reproducible causative knowledge that can then be extended to complex natural systems. While dynamical differences are indeed expected between in vitro and in vivo experiments, our work illustrates that generalization should come from an appropriate integration and interpretation of theoretical and experimental systems. Although not exhaustive, our work has provided a roadmap for this integration. In particular, our findings have revealed that structuralist-like theories may be a better companion for in vivo experimental systems when aiming to approximate the behavior of systems under natural changing conditions. Lastly, while it is outside the scope of our study, it is worth mentioning that in order to move from the phenomenological analysis presented in this work to a mechanistic understanding of microbial dynamics, it is essential to understand how differences between communities can be additionally driven by trait-based, resource-based, genome-based, or bottom-up assembly processes.

## Supplementary information

Supplementary material

## Data Availability

The code supporting the results can be found at https://github.com/MITEcology/ISMEC_Deng_etal_2021.
