# Supplementary material for: Generalizing game-changing species across microbial communities
Source: ISME Commun. 2021 Jun 8;1:22. doi: 10.1038/s43705-021-00022-2 (PMC9723773; doi:10.1038/s43705-021-00022-2)
Supplement: Supplementary file 1 — Supplementary material [file 43705_2021_22_MOESM1_ESM.pdf]

Supplementary Material for

Generalizing game-changing species across microbial communities

Jie Deng[1], Marco Tulio Angulo[2], Serguei Saavedra[1]

[1]Department of Civil and Environmental Engineering, MIT,
77 Massachusetts Av., 02139 Cambridge, MA, USA
[2]CONACyT - Institute of Mathematics, Universidad Nacional Autónoma de México,
Juriquilla, QRO, México

Contents

S1 Experimental systems **S2**

S2 Theoretical systems **S4**

S1 Experimental systems

S1.1 *In vitro* soil communities

In vitro soil communities were compiled from Ref. [30]: experiments were performed by co-inoculating species at varying initial fractions, and propagating them through five growth–dilution cycles. The overall interaction time was chosen such that species extinctions would have sufficient time to occur, while new mutants would typically not have time to arise and spread. The abundances are measured by calculating the optical density of the cultures as well as plating on solid agar media and counting colonies, which are unique to each species. All experiments were carried out in duplicate. To determine species extinction between a given set of species, the two replicates from the initial conditions were combined and classified as extinct any species whose median abundance was less than 1%, which was just above the limit of detection. For the readers' convenience, below we provide information about the species, media, competition experiments, and measurements as written in Ref. [30], who performed the experiments.

Species and media: The eight soil bacterial species used in this study were *Enterobacter aerogenes* (Ea, ATCC#13048), *Pseudomonas aurantiaca* (Pa, ATCC#33663), *Pseudomonas chlororaphis* (Pch, ATCC#9446), *Pseudomonas citronellolis* (Pci, ATCC#13674), *Pseudomonas fluorescens* (Pf, ATCC#13525), *Pseudomonas putida* (Pp, ATCC#12633), *Pseudomonas veronii* (Pv, ATCC#700474) and *Serratia marcescens* (Sm, ATCC#13880). All species were obtained from ATCC. The base growth media was M9 minimal media, which contained 1× M9 salts (Sigma Aldrich, M6030), 2 mM $MgSO_4$, 0.1 mM $CaCl_2$, 1× trace metals (Teknova, T1001). For the final growth media, the base media was supplemented with 1.6 mM galacturonic acid and 3.3 mM serine as carbon sources, which correspond to 10 mM of carbon for each of these substrates. These carbon sources were chosen from a set of carbon sources commonly used to characterize soil microorganisms (Biolog, EcoPlate) to ensure that each of the eight species survives in monoculture. Nutrient broth (0.3% yeast extract, 0.5% peptone) was used for initial inoculation and growth before experiments. Plating was done on 10 cm Petri dishes containing 25 ml of nutrient agar (nutrient broth with 1.5% agar added).

Competition experiments: Frozen stocks of individual species were streaked out on nutrient agar Petri plates, grown at room temperature for 48 h and then stored at 4 °C for up to two weeks. Before competition experiments, single colonies were picked and each species was grown separately in 50 ml Falcon tubes, first in 5 ml nutrient broth for 24 h and next in 5 ml of the experimental M9 media for 48 h. During the competition experiments, cultures were grown in Falcon flat-bottom 96-well plates (BD Biosciences), with each well containing a 150 μl culture. Plates were incubated at 25 °C without shaking, and were covered with a lid and wrapped in Parafilm. For each growth–dilution cycle, the cultures were incubated for 48 h and then serially diluted into fresh growth media by a factor of 1500. Initial species mixtures were prepared by diluting each species separately to an optical density (OD) of 3×10^{-4}. Different species were then mixed by volume to the desired composition. This mixture was further diluted to an OD of 10^{-4}, from which all competitions were initialized. For each set of competing species, competitions were conducted from all the initial conditions in which each species was present at 5%, except for one more abundant species. For example, for each species pair there were two initial conditions with one species at 95% and the other at 5%, whereas for the eight species competition there were eight initial conditions each with a different species at 65% and the rest at 5%.

Measurement of cell density and species fractions: Cell densities were assessed by measuring OD at 600 nm using a Varioskan Flash plate reader. Relative abundances were measured by plating on nutrient agar plates. Each culture was diluted by a factor between 105 and 106 in phosphate-buffered saline, depending on the culture's OD. For each diluted culture, 75 μl was plated onto an agar plate. Colonies were counted after 48 h incubation at room temperature. A median number of 85 colonies per plate were counted.

S1.2 *In vivo* gut communities

In vivo gut communities were compiled from Ref. [34]: experiments were performed by co-inoculating species overnight and maintaining bacterial colonization through frequent ingestion in different flies. Species collections were assessed by visually scoring and enumerating species using a standard curve. We classified as extinct any species whose relative abundance was less than 10% in at least 71% of all (47 to 49) replicates, which corresponds to less than 1% of cases under a binomial distribution with $p = 0.5$. For the readers' convenience, below we provide information about the species, media, competition experiments, and measurements as written in Ref. [34], who performed the experiments.

Fly stock maintenance: Wolbachia-free *Drosophila melanogaster* Canton-S flies were reared on cornmeal-based medium (6.67% cornmeal, 2.7% active dry yeast, 1.6% sucrose, 0.75% sodium tartrate, 0.73% ethanol, 0.68% agar, 0.46% propionic acid, 0.09% methylparaben, 0.06% calcium chloride, and 0.01% molasses). Fly stocks were maintained at 25 °C, 60% humidity, and 12:12 h light:dark cycles. Fly stocks were tested for the presence of known RNA viruses by RT-PCR and were virus-free. Germfree fly stocks were kept in sterile conditions over multiple generations to reduce heterogeneity due to parental nutrition derived from microbiome variability.

Germ-free fly preparation: Wolbachia-free and virus-free *Drosophila melanogaster* Canton-S flies reared on a cornmeal-based medium were transferred to embryo collection cages and allowed to acclimate in the cage for at least one day before egg collection. On the morning of egg collection, a yeast paste was added on a grape juice agar plate. Flies were left to lay eggs on this grape juice agar plate for 5-6 h. Eggs were then collected into a 400 μm cell strainer. In a biosafety cabinet, fly eggs were rinsed twice in 10% bleach (0.6% sodium hypochlorite) for 2.5 min each, once in 70% ethanol for 30 s, and three times in sterile dH_2O for 10 s each. Approximately 50 eggs were transferred using a sterile cotton swab to individual vials containing sterile fly medium (10% filter-sterilized glucose, 5% autoclaved active dry yeast, 1.2% autoclaved agar, 0.42% filter sterilized propionic acid). The resulting adults were maintained germ-free for at least three generations to mitigate any parental effects.

Bacteria strains: Five unique species were identified: *Lactobacillus plantarum*, *L. brevis*, *Acetobacter pasteurianus*, *A. tropicalis*, and *A. orientalis*, which were then isolated from *D. melanogaster* flies and checked by standard Sanger sequencing the complete 16S region. To prepare the inoculum for flies, bacteria were grown overnight in MRS medium in a shaker set at 30 °C. The bacteria were resuspended at a concentration of 10^8 cells/mL in sterile phosphate-buffered saline (PBS) for fly gnotobiotic preparations so that constant numbers of CFUs were inoculated per fly vial. The 32 combinations of the 5 bacterial strains were mixed using a Beckman Coulter Biomek NXP workstation to standardize the inoculum. Vials were swabbed to ensure correct bacterial species were present without contaminants.

Adult gnotobiotic fly preparation: Germ-free mated flies 5-7 days post-eclosion were sorted into 10% glucose, 5% active dry yeast medium inoculated with a defined mixture of bacteria. Flies thus treated have been shown to have less variation in physiology and gut morphology. Inoculating in the adult phase allowed us to separate developmental differences from effects on adult flies. Each vial contained a total of 5×10^6 CFUs (50 μL of 10^8 bacteria/mL in 1× PBS). Ten female and ten male flies were transferred into each vial. Gnotobiotic flies were transferred to freshly inoculated medium every 3 days for the duration of the concurrent lifespan-fecundity-development experiment.

Bacterial ecology calculations: Two different treatments were applied after an initial 10 days of inoculation where flies were inoculated. Every three days the remaining live flies were transferred to fresh food vials inoculated with $5{\times}10^6$ CFUs/vial (as with the initial inoculation) in order to reduce the effects of microbial interactions on the fly media. Vial swabs were performed to check

that all inoculated species were still present on the third day. In the first treatment (n=24 flies per bacterial treatment), flies were immediately subjected to bacterial load quantification on the 10th day of inoculation. This experiment evaluates the load and relative abundance of bacteria during the fly fitness experiment. A second treatment was undertaken to measure the steady state of bacterial population sizes in the absence of new colonization. After the initial 10 days of inoculation, these flies were transferred daily to fresh germ-free food for 5 days before subsequent bacterial load quantification.

S2 Theoretical systems

S2.1 Structuralist framework

To carry out our structuralist analysis, we consider a community of S species. We assume that the community structure and dynamics can be described by any ecological model topologically equivalent to the linear Lotka-Volterra (LV) system, including extensions to nonlinear functional responses and stochastic functions [32, 48]:

$$\frac{\mathrm{d}N_i}{\mathrm{d}t} = N_i\Big(r_i + \sum_{j=1}^{S} a_{ij}N_j\Big). \tag{S1}$$

Above, N_i is the abundance (or biomass) of species i, r_i represents the intrinsic growth rate of species i, and $\mathbf{A} = (a_{ij})$ is the interaction matrix representing the per-capita effect of species j on an individual of species i (the biotic context). The linear LV system in Eq. (S1) is a phenomenological model written in the r-formalism, implying that intrinsic growth rates represent the summary effect of external conditions on the per capita growth of populations.

The feasible interior equilibrium of Eq. (S1) is $\mathbf{N}^* = -\mathbf{A}^{-1}\mathbf{r}$, where $\mathbf{N}^* = (N_1^*, \dots, N_S^*)^\top$ ($N_i^* > 0$) is the vector of abundances at equilibrium, $\mathbf{r} = (r_1, \dots, r_S)^\top$ is the vector of intrinsic growth rates, $\mathbf{A}^{-1}$ is the inverse of the interaction matrix. That is, for a given interaction matrix $\mathbf{A}$, feasibility in Eq. (S1) will be satisfied as long as the $\mathbf{r}$-vector falls inside the feasibility domain $D_F(\mathbf{A}) = \{N_1^* > 0, \dots, N_S^* > 0 \text{ such that } -N_1^*\mathbf{a}_1 - \dots - N_S^*\mathbf{a}_S = \mathbf{r}\}$, where $\mathbf{a}_i$ is the i-th column vector of the interaction matrix $\mathbf{A}$ [40]—this corresponds to the match between internal and external conditions (Figure 2 Panel F dark symbols). Because the feasibility domain is described by the positive linear combinations of the columns of matrix $\mathbf{A}$, it corresponds to an algebraic cone in $\mathbb{R}^S$ [37, 40]. This geometric view of the feasibility domain leads to a probabilistic interpretation. The size of the feasibility domain is equivalent to the probability of randomly sampling a direction of the $\mathbf{r}$-vector that falls inside the algebraic cone defined by $D_F(\mathbf{A})$.

To compute the normalized size of the feasibility domain, we first define the unit ball in $\mathbb{R}^S$ as $\mathbb{B}^S = \{\mathbf{u} \in \mathbb{R}^S, \|\mathbf{u}\|_2 \leq 1\}$. Then, the normalized size of the feasibility domain (i.e., probability of feasibility) can be computed as the volume of the algebraic cone that is inside the unit ball divided by the volume of the unit ball inside the positive quadrant $\mathbb{R}^S_{>0}$:

$$\Omega(\mathbf{A}) = \frac{2^S \mathrm{vol}(D_F(\mathbf{A}) \cap \mathbb{B}^S)}{\mathrm{vol}(\mathbb{B}^S)}, \tag{S2}$$

where 2^S normalizes the feasibility domain to positive parameter values (i.e., $r_i > 0 \ \forall \ i$). Thus, $\Omega(\mathbf{A}) = P(\mathbf{r} \in D_F(\mathbf{A})) \in [0, 1]$, where the upper bound represents a scenario with no interspecific interactions. Assuming that any direction of the $\mathbf{r}$-vector is equally likely, the size of the feasibility domain can be numerically estimated by solving the following integral [60]:

$$\Omega(\mathbf{A}) = 2^S \frac{\mathrm{vol}(D_F(\mathbf{A}) \cap \mathbb{B}^S)}{\mathrm{vol}(\mathbb{B}^S)} \tag{S3}$$

$$= 2^S \frac{\det(\mathbf{A})}{\sqrt{\pi^S}} \int_{\mathbb{R}^S_{\geq 0}} e^{-\frac{1}{2}\mathbf{N}^{*\top}\mathbf{A}^\top\mathbf{A}\mathbf{N}^*} d\mathbf{N}^* \tag{S4}$$

$$= 2^S \frac{1}{\sqrt{(2\pi)^S \det(\mathbf{\Sigma})}} \int_{\mathbb{R}^S_{\geq 0}} e^{-\frac{1}{2}\mathbf{N}^{*\top}\mathbf{\Sigma}^{-1}\mathbf{N}^*} d\mathbf{N}^* \tag{S5}$$

where we set $\mathbf{A}^\top\mathbf{A} = \mathbf{\Sigma}^{-1}$ in order to obtain the cumulative distribution function of a multivariate normal distribution with mean $\mu = 0$ and covariance matrix $\mathbf{\Sigma}$. Equation (S3) can be computed efficiently for even relatively large communities by solving numerically the integration using a quasi-Monte Carlo method [40, 61].

By assuming that the sampling of the directions of intrinsic growth rates is independent and identically distributed for every species, we can compute the probability that a randomly chosen species i is feasible (i.e., $N_i^* > 0$) as [32, 40]:

$$p(i|\mathbf{A}) = \Omega(\mathbf{A})^{\frac{1}{S}} \tag{S6}$$

This species-level measure is bounded as $p(i|\mathbf{A}) \in [0, 1]$ and can be interpreted as the probability of feasibility under changing environments. Note that the intersection of the two feasibility domains $D_F(\boldsymbol{A})$ and $D_F(\boldsymbol{B})$ (the positive orthant of the unit ball) can then be mathematically formalized as the linear combination $\sum N_i^* \boldsymbol{a}_i$ of the spanning vectors of $\boldsymbol{A}$, where N_i^* satisfies

$$\begin{cases} \sum_{i=1}^{S} N_i^* \boldsymbol{a}_i = \sum_{i=1}^{S} \mu_i \boldsymbol{b}_i \\ N_i^*, \mu_i \geq 0, i = 1, .., S. \end{cases}, \tag{S7}$$

and $\boldsymbol{a}_1, .., \boldsymbol{a}_S$ and $\boldsymbol{b}_1, .., \boldsymbol{b}_S$ are the spanning vectors of the cone $D_F(\boldsymbol{A})$ and $D_F(\boldsymbol{B})$, respectively. Then, we need to apply the triangularization of the intersected region into several cones, and the intersected volume can be computed by adding the volume of each cone together [40].

S2.2 Inferring the interaction matrix

The dataset we analyzed collected by Gould *et al.* [34] consists of $S = 5$ species, enumerated as follows:

1. *Lactobacillus plantarum*, 2. *Lactobacillus brevis*, 3. *Acetobacter pasteurianus*, 4. *Acetobacter tropicalis*, 5. *Acetobacter orientalis.*

The dataset contains the equilibrium absolute abundance measured in Colony Forming Units (CFUs) for each of the $2^5 - 1 = 31$ different communities that can be assembled. Each species collection has between 40 and 49 replicates. The goal is to obtain an estimate $\hat{A} = (\hat{a}_{ij}) \in \mathbb{R}^{S\times S}$ of the interaction matrix $A = (a_{ij}) \in \mathbb{R}^{S\times S}$ of the linear Lotka-Volterra (LV) system of Eq. (S1). Here, a_{ij} is the effect of species j on the per-capita growth rate of species i.

To infer the interaction matrix, we consider a subset of the above dataset containing only one or two species. Therefore, the available information is the abundance of species i in isolation, and how its abundance changes when species j is introduced. A small change in the abundance of species i due to the introduction of species j is evidence of a negligible a_{ij}, such as when species

2 is added to species 1 (Fig. S1A). By contrast, a large change in abundance is evidence of a significant a_{ij}, such as when species 1 is added to species 2 (Fig. S1B).

To quantify the value of a_{ij}, let x_i^* denote the equilibrium abundance of the i-th species in isolation. Let (y_i^*, y_j^*) denote the equilibrium abundance of species i and j when both are present. Then, for all data such that $x_i^* \neq 0$ and $y_i^* \neq 0$, the LV system of Eq. (S1) implies that the following equations must be satisfied:

$$\begin{aligned} r_i + a_{ii}x_i^* &= 0, \\ r_i + a_{ii}y_i^* + a_{ij}y_j^* &= 0. \end{aligned} \tag{S8}$$

Note that, since only steady-state measurements are available, there are fundamental limitations in the information of a_{ij} that can be inferred [62]. Namely, in the above equations we have three unknown (r_i, a_{ii}, a_{ij}) but only two equations, implying there is no sufficient information to constraint the three unknowns. To circumvent this limitation, we can assume $\hat{a}_{ii} = -1$ without loss of generality [63]. With this assumption, and subtracting the first from the last line of Eq. (S8), we obtain

$$a_{ij} = \frac{y_i^* - x_i^*}{y_j^*}, \tag{S9}$$

which can be used to estimate a_{ij} from the experimental data. Importantly, since there are several replicates for x_i^* and (y_i^*, y_j^*), Eq. (S9) actually characterizes a distribution of possible values for a_{ij} obtained by taking an arbitrary pair of replicates. To obtain the single value necessary for our predictions, we defined

$$\hat{a}_{ij} = \text{Median}(a_{ij}), \quad i \neq j,$$

estimated using a Bootstrap method with 100,000 pairs.

To illustrate the inference process, Figure S1C shows the distribution obtained for a_{12}. Calculating the median value gives $\hat{a}_{12} = 0$. This result is consistent with the fact that species 2 produces little change in the abundance of species 1, as observed in Fig. S1A. By contrast, the distribution for a_{21} is charged to negative values with $\hat{a}_{21} = -1.119$ (Fig. S1D). This result is consistent with the fact that species 1 decreases the abundance of species 2, as observed in Fig. S1B. By repeating this process for all pairs of species, we obtained the following estimate for the interaction matrix:

$$\hat{A} = \begin{pmatrix} -1 & 0 & 0.182 & 0.091 & 0.197 \\ -1.119 & -1 & -0.097 & -0.34 & -0.094 \\ 0.592 & 0 & -1 & 0 & 0 \\ -0.288 & -0.036 & 0 & -1 & -0.576 \\ 0 & 0 & 0 & -0.239 & -1 \end{pmatrix}. \tag{S10}$$

To homogenize our structuralist analysis across *in vitro* and *in vivo* communities, we inferred the interaction matrix of the soil communities in the same way as we did for the gut communities (as mentioned above) using the data reported in the supplementary information of Ref. [30]. Note that in the original study [30], the interaction matrix was inferred by fitting the linear LV system to the data using a least-squares method. We also ran our analysis using the originally inferred matrix and results presented in Fig. 4 (main text) were qualitatively similar. Our inferred matrix

for the *in vitro* communities using median values is:

$$\hat{A} = \begin{pmatrix} -1 & -0.75 & -1 & -0.283 & -1.311 & -0.928 & -1.04 & 0 \\ 1 & -1 & -0.571 & 0.999 & -0.75 & -0.211 & 0.533 & -0.272 \\ 0.375 & 2 & -1 & 0 & 0.499 & 0.333 & 0.214 & 0 \\ 0.344 & 0 & 0 & -1 & 6.5 & 0 & 0 & 0.4 \\ 0.1 & -0.375 & -0.5 & -0.6 & -1 & 0.045 & 0.04 & -0.055 \\ -0.999 & -1.176 & -0.681 & 0.5 & -0.666 & -1 & 1 & 0.5 \\ 0.5 & -0.409 & -0.5 & -0.499 & 0.083 & -0.687 & -1 & 0 \\ -1.035 & -1.818 & -1 & -1.1 & -1.3 & -0.935 & -0.875 & -1 \end{pmatrix}. \quad \text{(S11)}$$

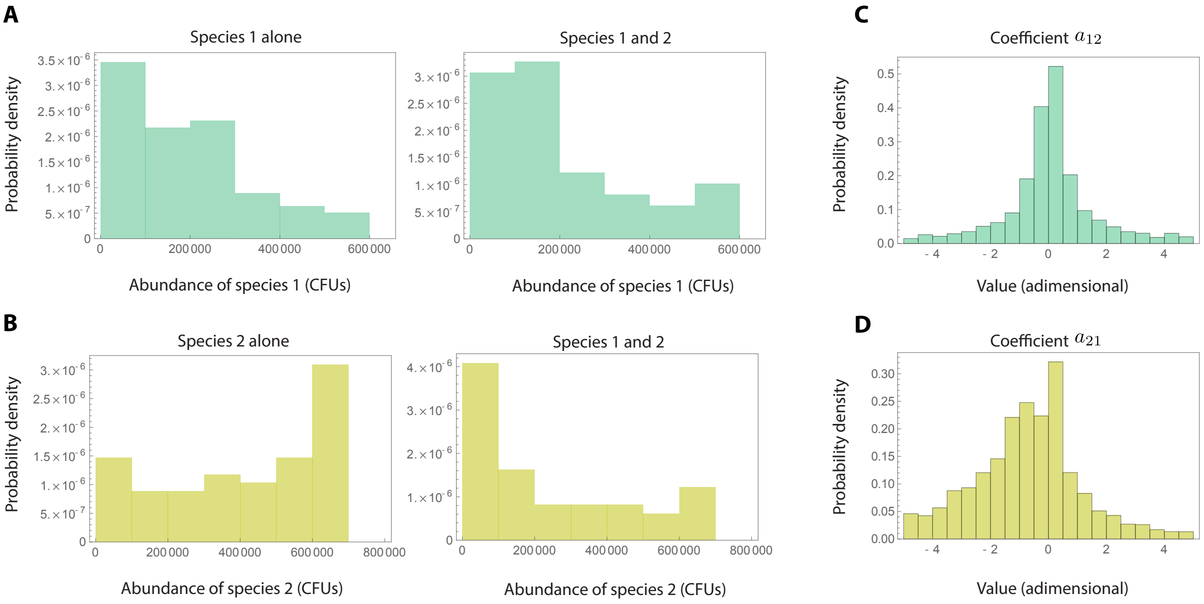

Supplementary Figure S1: Inferring the interaction matrix from experimental data of *Drosophila melanogaster* gut microbiota.
